# Simultaneous DNA-based diet analysis of breeding, non-breeding and chick Adélie penguins

**DOI:** 10.1098/rsos.150443

**Published:** 2016-01-13

**Authors:** Julie C. McInnes, Louise Emmerson, Colin Southwell, Cassandra Faux, Simon N. Jarman

**Affiliations:** Australian Antarctic Division, Channel Highway, Kingston, Tasmania, Australia

**Keywords:** faecal, molecular, food, next-generation sequencing

## Abstract

As central place foragers, breeding penguins are restricted in foraging range by the need to return to the colony to feed chicks. Furthermore, breeding birds must balance energetic gain from self-feeding with the costs of returning to provision young. Non-breeding birds, however, are likely to be less restricted in foraging range and lack the high energy demands of provisioning, therefore may consume different prey to breeders. We used DNA dietary analysis to determine whether there was a difference in provisioning and self-feeding diet by identifying prey DNA in scat samples from breeding and chick Adélie penguins at two locations in East Antarctica. We also investigated diet differences between breeders and non-breeders at one site. Although previous work shows changing foraging behaviour between chick provisioning and self-feeding, our results suggest no significant differences in the main prey groups consumed by chicks and breeders at either site or between breeding stages. This may reflect the inability of penguins to selectively forage when provisioning, or resources were sufficient for all foraging needs. Conversely, non-breeders were found to consume different prey groups to breeders, which may reflect less restricted foraging ranges, breeders actively selecting particular prey during breeding or reduced foraging experience of non-breeders.

## 1. Introduction

Many animals are restricted in their foraging range during breeding, as they must return to a central place such as a nest or den. This limitation placed on central place foragers means that individuals must determine the optimal foraging strategy to maximize patch time and prey selection [1]. For breeding birds, partitioning of food resources between self-feeding and provisioning reflects a compromise between inclusive fitness of the parent and its offspring and immediate energetic gain [2,3]. Most seabird species are central place foragers during the breeding season as they must return to their nest to incubate eggs and provision young, with a large proportion of species breeding colonially. In many seabirds, exploitation of prey in close proximity to the breeding colonies increases as the food demands of the chicks increase, decreasing food availability near the colony [4,5]. This ‘halo effect’ means that parents are required to forage further from the colony, restricting their flexibility in foraging patch selection, increasing the foraging trip duration and therefore increasing the cost of provisioning. Conversely, non-breeding individuals are less constrained by foraging location and prey, allowing them to move further from the colony and consume a more diverse diet [6,7].

Different diet analysis methods are often required to identify the diet of different age and breeding classes. For some short-ranging seabird species that carry whole prey in their bills, observational studies of self-feeding and provisioning may be achievable [8,9]. When direct observations are not possible, dietary comparisons of prey are difficult because different techniques may be required to identify the prey of different cohorts within the population [10,11]. The two main diet sampling techniques used for these studies are hard-part analysis of stomach contents and stable isotope analysis. Stable isotope analysis measures the C13/C12 ratios and N15/N14 ratios of feathers or blood and allows broad-scale dietary comparisons across latitudinal gradients or trophic levels [12]. This technique has successfully been used to compare the trophic level of different cohorts of a population [13], but lacks taxonomic resolution because the composition of prey types in the diet cannot be identified. To identify specific prey consumed, regurgitated stomach content samples are taken either from chicks [14] or from breeding adults returning to the colony to feed chicks [15]. Therefore, only the prey that parents provision their chicks is identified rather than what the parent is digesting itself.

Molecular analysis of faecal DNA can identify prey sequences in predator scats [16], and from this we can identify the relative proportion of each prey group [17]. This does not necessarily equate to biomass [18], however comparison of relative proportions is a widely accepted approach in molecular studies [19,20]. DNA dietary analysis is becoming a popular tool as it allows comparisons between breeding stages, colonies [21] and cohorts [22], making it a useful alternative diet analysis method. Importantly, this dietary tool allows us to identify any differences between the diet of adults that are provisioning offspring compared with their diet when self-feeding.

Adélie penguins are an ideal species to study diet differences between cohorts. The breeding population of this Antarctic species has a restricted foraging range during the breeding season due to the need to return to feed chicks. Furthermore, their foraging effort increases with colony size as suggested by the ‘halo’ theory [23]. Throughout the breeding season, Adélie penguins can alter their foraging behaviour [24] and diet [21,25]. Chicks require constant parental care while small (guard period) until they can be left unguarded (crèche period) [26]. During the period when the chicks are young and require frequent meals, the parents alternate between nest attendance and foraging. Because at this stage parents forage primarily for the chick, they can lose body condition until the chicks reach ‘crèche’ stage and the parents are able to forage for self-maintenance [24]. However, as they provision chicks by regurgitation, variation between chick and adult diet is difficult to study.

Non-breeding penguins are rarely the focus of dietary studies during the breeding season, even though in some species a large number of non-breeding birds may be present in the colony, and certainly this is the case for Adélie penguins [26].

This study uses a DNA-based diet approach to identify if there are any differences in the proportion of major prey groups consumed by breeding and chick Adélie penguins at two sites in east Antarctica, and breeding and non-breeding birds at one of these sites. No penguin diet study to our knowledge has compared breeder and non-breeder diet during the breeding season or simultaneously sampled the prey consumed by breeders and chicks.

## 2. Material and methods

### 2.1 Sample collection

Adélie penguin faecal samples were collected in the 2012/2013 austral summer in East Antarctica at two breeding colonies 2000 km apart: Béchervaise Island, 3 km northwest of Mawson Station (67°35′ S, 62°49′ E); and Whitney Point, 7 km from Casey Station (66°16′ S, 110°31′ E). Penguins were observed until they defecated, at which point a small fragment of the non-uric acid portion of the scat was collected using tweezers and stored in 80% ethanol. The samples were kept as cool as possible after collection and stored at −20°C during transport from Antarctica. Samples were collected during two stages of the breeding season: brood guard (Béchervaise Island 4–7 January 2013, Whitney Point 23–28 December 2012) and mid-crèche (23–26 January 2013). Scat samples were collected from breeding birds, chicks and non-breeders at Béchervaise Island and breeding birds and chicks at Whitney Point. ‘Breeders’ were identified as individuals brooding or provisioning a chick, whereas ‘non-breeders’ were usually pairs that had reoccupied the colony and were building new practice nests with no chick present. Non-breeders in the colony include immature birds that have not yet bred and mature birds of breeding age that did not breed in a particular season (e.g. no partner or insufficient body condition). Samples were collected randomly throughout the colony across a 4–6 day time period to ensure that any short-term changes in the diet at the population level would be incorporated; however, it still represents a discrete time period with which to compare to another breeding stage. The digestion rates of different cohorts are largely unknown for this species; however, we believe this is unlikely to be an issue as the time period of collection would have represented multiple foraging trips across the population, as trip duration was generally less than 4 days. Captive trials, using little penguins (*Eudyptula minor*), found that prey could be detected for up to 4 days after ingestion [18].

### 2.2 DNA extraction and DNA metabarcode amplification

DNA amplification and sequencing of samples were carried out as described by Jarman *et al.* [21]. In brief, DNA was extracted from faecal samples using a Promega ‘Maxwell 16’ instrument and a Maxwell^®^ 16 Tissue DNA Purification Kit. A DNA region of approximately 140 bp from the 3′ end of the nuclear small subunit ribosomal RNA gene was amplified with primers that are conserved among most eukaryotes (primers available in Jarman *et al.* [21]). PCR inhibitor concentrations were reduced in the DNA extracts by a Zymogen ‘One Step^TM^ PCR Inhibitor Removal’ kit. PCR reactions (10 μl) were performed with 5 μl 2 × Phusion HF (New England Biolabs), 1 μl 100 × bovine serum albumin (New England Biolabs), 1 μl of each 1 μM amplification primer [21], 1 μl of 10 μM blocking primer ‘TetrapodBlockc3’ and 1 μl faecal DNA . Thermal cycling conditions were 98°C for 2 min, followed by 35 cycles of 98°C for 5 s, 55°C for 20 s, 72°C for 20 s. PCR reactions from each set were pooled and purified from unincorporated reaction components by washing using reversible binding to Ampure (Agencourt) magnetic beads following the manufacturer's protocol.

### 2.3 High-throughput amplicon sequencing

Sequencing of PCR products was performed with an IonTorrent next-generation sequencer and OneTouch semi-automated library preparation platform (Life Technology), using the 400 bp sequencing kit v1 and 314 chips. Primary sequence estimation was done by Torrent Server software version 2.2 with the ‘Beverly read filter’ turned off to ensure the maximum number of sequences were included in the primary data. FASTQ files were transferred from the Torrent Server after checking run success based on the run reports generated by the Torrent Suite software. Samples from each location and cohort were split among different runs to avoid run-specific biases.

### 2.4 Sequence data processing

FASTQ files were first filtered by read quality with those having a mean *Q*-score less than 30 removed. Sequences were then grouped into molecular operational taxonomic units (MOTUs) of more than 0.9 sequence similarity using USEARCH [27]. A 90% cut-off of MOTUs was used to allow taxonomic discrimination between prey groups to family level. Higher level taxonomic assignment was not possible for most of these broad groups because of limited variation in the ‘V9 region’ of the of the SSU rRNA gene. Each MOTU was identified by BLAST against a local database derived from the SILVA ssu database release 118 [28]. Non-food sequences such as parasites and contaminants were excluded from analysis. Any samples containing less than 100 food sequences were excluded from the analysis. The proportion of food sequences for each cohort was calculated by averaging the sequence proportions for each individual scat.

### 2.5 Analysis and statistics

**Figure 1.**
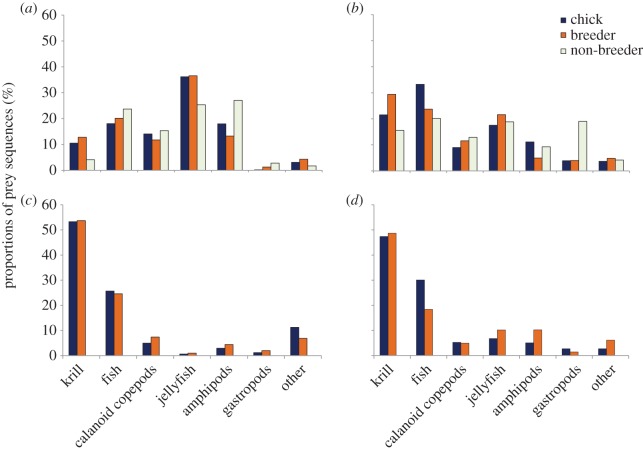
Adélie penguin prey proportions. Average proportion of sequences for the main six prey groups found in Adélie penguin scats in East Antarctica. The category ‘other’ contained prey sequences that represented less than 2% of the overall sequences. Comparisons between chicks, breeder and non-breeders at Béchervaise Island during (*a*) chick-guard and (*b*) crèche; and for chicks and breeders at Whitney Point during (*c*) chick-guard and (*d*) crèche.

Overall there were 27 prey taxa identified in the samples (electronic supplementary material, table S1). For comparisons of prey between cohorts, prey groups included were those that had a proportion of total sequences greater than 2%. Prey groups with less than 2% of sequences were combined into the group ‘other’. Six prey groups had more than 2% of sequences and were used in the analysis (figure 1). Analysis of similarity (ANOSIM) tests were carried out using the statistical package R to determine whether there were differences between the proportions of the six groups between cohorts. Data were fourth log transformed to improve normality. Simper analysis was used to identify which components of the diet caused any observed differences. To identify the diversity of prey consumed, the Shannon diversity index was used to estimate taxa diversity. This analysis incorporated all 27 taxa found across sites (see table 1; the electronic supplementary material). Where no differences were found between cohorts, samples were pooled to describe the diet at each breeding site. The Shannon diversity index is: $H^{\prime }=-\sum p_{i}$ ln *p*_*i*_, where *p*_*i*_ is the proportion of sequences belonging to the *i*th taxa groups.

**Table 1.** The number of samples collected, DNA extracted and food samples amplified with more than 100 DNA sequences for each site, breeding stage and breeding cohort.

site	stage	cohort	samples collected	samples more than 100 food sequences	proportion successful
Béchervaise Island	chick-guard	chick	60	55	91.7
		breeder	74	54	73.0
		non-breeder	55	35	63.6
	crèche	chick	60	47	78.3
		breeder	64	33	51.6
		non-breeder	60	36	60.0
Whitney Point	chick-guard	chick	19	15	78.9
		breeder	35	24	68.6
	crèche	chick	33	24	72.7
		breeder	33	25	75.8
		total	493	348	70.6

## 3. Results

### 3.1 Sample collection and amplification success

Four hundred and ninety three scat samples were collected during chick-guard (December 2012) and crèche (January 2013), with 373 collected from Béchervaise Island and 120 collected at Whitney Point. In total, 70.6% of samples successfully amplified over 100 prey sequences each, allowing 348 samples to be analysed (table 1).

### 3.2 Breeder and chick prey

No significant difference was observed among the proportion of prey sequences for breeders or the proportion for chicks at Béchervaise Island (chick-guard: *r*=−0.008, *p*=0.784; crèche: *r*=−0.007, *p*=0.575) or Whitney Point (chick-guard: *r*=−0.005, *p*=0.403; crèche: *r*=−0.007, *p*=0.530; figure 1 and table 2). When chick and adult samples were pooled, scyphozoa (jellyfish) were the most prevalent prey item found in chick and breeder scats at Béchervaise Island during chick-guard, representing 36% of prey sequences. The remaining prey sequences included 11–19% each of actinopterygii (bony fish), euphausiids (krill), amphipods and calanoid copepods. As the breeding season progressed, there was an increase in bony fish from 19 to 29% and krill from 12 to 25%, and a decrease in jellyfish from 36 to 19% (guard versus crèche stages). At Whitney Point, the prey sequence proportions were similar between the breeding stages, with krill and fish sequences dominating the scats (figure 1).

**Table 2.** Analysis of similarities (ANOSIM) of diet proportions for each breeding location and stage between (*a*) breeders and chicks and (*b*) breeders and non-breeders. Sample sizes represent the number of samples that amplified over 100 prey sequences. Asterisk indicates significant difference.

site	stage	*n* breeder	*n* chick	*R*	*p*-value
(*a*)					
Béchervaise Island	chick-guard	54	55	−0.008	0.784
	crèche	33	47	−0.007	0.575
Whitney Point	chick-guard	24	15	−0.005	0.403
	crèche	25	24	−0.007	0.530

site	stage	*n* breeder	*n* non-breeder	*R*	*p*-value
(*b*)					
Béchervaise Island	chick-guard	54	35	0.062	0.025*
	crèche	33	36	0.033	0.069

### 3.3 Breeder and non-breeder prey

There were differences observed in the proportion of prey sequences of breeders and non-breeders at Béchervaise Island during chick-guard (*r*=0.062, *p*=0.025), but no significant difference during crèche (*r*=−0.033, *p*=0.069; table 2). During chick-guard, non-breeders' scats had less jellyfish and more amphipod sequences than breeders, whereas during crèche there were less krill and jellyfish sequences in the non-breeder scats and more gastropods (figure 1).

### 3.4 Prey diversity

At Béchervaise Island, there was an increase in diversity between the chick-guard and crèche (*F*_1,254_=6.691, *p*=0.01), but there was no difference between cohorts (*F*_1,254_=0.709, *p*=0.4929) and the interaction term between breeding stage and cohort was not significant (*F*_2,254_=2.673, *p*=0.071). At Whitney Point, there was no difference in diversity between breeding stages (*F*_1,112_=1.030, *p*=0.312) or cohorts (*F*_1,112_=0.609, *p*=0.546) and the interaction term between breeding stage and cohort was also not significant (*F*_2,112_=0.537, *p*=0.586).

## 4. Discussion

Our study investigated the diet of Adélie penguins by simultaneously estimating the diet of breeding and chick Adélie penguins at two breeding populations separated by 2000 km in East Antarctica, as well as the diet of non-breeders at one of these sites. This comparison was made using DNA-based assessments of prey groups in penguin scats, allowing the diet of different cohorts within the same population to be compared. Breeders were found to provision chicks with similar prey to the prey they digested themselves, even with different resources available at the two different sites. In comparison, non-breeders were found to consume more gastropods and amphipods than breeders; however, the overall prey diversity between the groups was similar.

### 4.1 Breeder and chick diet

Diet similarities between chicks and breeding birds were found at both breeding sites, suggesting that parents are not altering their prey selection when provisioning chicks or self-feeding. The diet of penguins at Béchervaise Island was not dominated by any one of the six prey groups during either chick-rearing or crèche, whereas at Whitney Point the diet was dominated by krill. This allowed us to assess diet at two colonies with different prey availability or resource requirements.

It is difficult to assess diet quality based purely on energy content of prey items, as ingestion rates and foraging strategies may affect the prey selected or consumed. For example, Antarctic silverfish (*Pleuragramma antarcticum*), one of the main fish prey taken by Adélie penguins [29], has a higher calorific and lipid content than krill [30,31]. However, a previous study at Béchervaise Island has shown that high krill content in the penguins' diet is linked with higher breeding success, although these years also had larger meal masses, making the primary cause of high breeding success hard to delineate [25]. Likewise, jellyfish, which have low calorific and lipid content [32], formed a large proportion of the prey sequences at Béchervaise Island in this study, in a year that had an above average breeding success (0.76 chicks per occupied nest compared with an average of 0.68 between 1991/1992 and 2002/2003 [25]). The significance of jellyfish for Adélie penguin diet is poorly understood as gelatinous prey are difficult to identify in stomach contents, so past studies relying on stomach lavage have not considered this component. However, jellyfish are present in the diet of many other seabird species [33]. The role of jellyfish in seabird diet warrants further investigation to understand whether they are targeted prey items or ingestion is incidental, particularly given the expected shift towards more jellyfish under future environmental change [34].

At Whitney Point, it seems that krill and fish were relatively easy to access compared with at Béchervaise Island, and under those conditions we would expect the parents to forage on the same high-quality prey that they fed their chicks. At Béchervaise Island, both krill and fish were less frequent in the penguin scats, which is in contrast to our expectation that those two items would be in a higher proportion of the chick scats than those of the parents if they were returning higher quality prey to their offspring. This was not the case, suggesting that the breeders may not be selectively foraging for their chicks. There is ample evidence that Adélie penguins at Béchervaise Island alter their foraging strategy when self-feeding through either longer duration or more distant trips [24]. During chick-guard, foraging trips are relatively short and adult body mass declines suggesting parents forage primarily to provision young [35]. During crèche, foraging trips are longer and adult mass remains steady [35] even though greater foraging distances lead to increased energy expenditure [23]. This indicates parents have increased self-feeding as both males and females can forage simultaneously, reducing the frequency of provisioning requirements. Previous analysis of stable isotopes during crèche revealed parents were likely to be feeding offshore for themselves and inshore for chicks [36]. However, our results indicate no feeding difference between chicks and breeders, and previous work with stable isotope comparison between chicks and breeders showed no difference in trophic level [36]. Intraspecific competition in close proximity to some Adélie penguin breeding colonies may be high and may increase throughout the breeding season as resources decline [23]. Although provisioning a chick with prey caught inshore is presumably beneficial because of the reduced costs and time associated with foraging locally, if intraspecific competition is high in this region then foraging further afield, at locations where prey can be reliably found [24], may be more beneficial for self-feeding, even if the prey is the same.

It is also possible that penguins cannot selectively control which prey items are regurgitated to their chick. Although differences have been found in trophic level between adults and chicks using stable isotopes [13,36,37], no penguin dietary studies have investigated stomach contents from both cohorts. Overall, there are few published studies in other seabird species that provision by regurgitation which analyse stomach contents of both chicks and adults. However, one study of albatross suggests that this is possible [38].

In this study, we taxonomically identified prey in groups of species to allow a wide taxonomic range of prey to be identified. This approach is technically useful for characterizing the full range of prey groups; however, there may have been variation between species or individuals within a species that were fed to chicks that could not be detected by this approach. Selective foraging is found in other seabirds that target different prey species within a group with higher lipid content, or choose larger or gravid individuals [8,39]. Although prey size cannot be detected with this technique, future studies could target a group-specific gene region to investigate whether species differences are present [22].

### 4.2 Breeder and non-breeder diet

Our study found a difference between the proportions of prey consumed by breeders and non-breeders during chick-guard. The diet of non-breeding birds has not been investigated during the breeding season for penguins and little is known about their behaviour during this period. In general, non-breeding seabirds are believed to be unconstrained by the need to return to the colony to provision chicks, therefore have the potential to forage for longer periods and at greater distances from the colony and obtain different prey than what breeders can obtain. However, because these non-breeders have returned to the colony either to establish a nest site or develop pair bonds [26], they may still have limits to their foraging range if they are tied to the colony for a period. A proportion of non-breeders returns to the colony for either short or extended periods depending on their age and sex [40]. The foraging range and diet flexibility of these non-breeding birds are unknown during this period.

The selection of certain prey groups could be related to requirements for breeding. Non-breeder energy demands may be much lower than those of breeders [41] and could be met with alternative prey, while limiting intraspecific feeding competition for limited resources. Conversely, breeders may need to increase the consumption of certain prey to meet the increased energy demands of provisioning young or to provide important nutrients for chick-rearing which non-breeders do not require. In Wilson's storm petrels, a comparison of diet between breeders and non-breeders over a longer period found a shift in breeders' diet at the start of breeding, then a return to the same diet as non-breeders at the end of the season [42]. This shift in diet was identified as a need to meet the nutritional requirements of the chicks with higher calcium prey. If this was the case in Adélie penguins, then this may explain why there was a greater diet difference between the two cohorts during chick-guard, when the provisioning rate is higher than self-feeding. If certain prey is required for birds to successfully breed, this may help partially explain why some of these birds did not breed that year. Alternatively, non-breeding birds may be inexperienced or poor foragers. Inexperienced seabirds may exhibit different foraging habitats or behaviours than experienced birds [43]. As non-breeders are often immature birds, foraging experience may not be as high as older birds.

As with most dietary analysis methods, there are potential biases that could be affecting our results. Possible biases may arise from differential digestion rates between age cohorts and the effect of DNA degradation in scats. It is possible that the observed difference between breeder and non-breeder prey sequences could be exacerbated if these biases did exist, however the fact that the prey sequences between chicks and adults was indistinguishable suggests that this bias is unlikely to be an issue in this study.

## 5. Conclusion

This study has simultaneously identified the diet of breeding, chick and non-breeding Adélie penguins for the first time. The prey groups selected during provisioning and self-feeding were similar at each site in 2012/2013 even though the diets between sites were different. Although previous work at Béchervaise Island has shown that foraging behaviour in terms of trip duration and length changes between provisioning chicks and self-feeding [24], our results showed no evidence of prey difference between these two cohorts. Breeding Adélie penguins consume and provision their chicks with different prey than that consumed by non-breeders. We suggest that this is due to the non-breeders having a less restricted foraging range and therefore having access to alternative prey. However, if they are also constrained to the colony and cannot access other food, they may be less selective because they have lower energy demands due to not provisioning, or they may be inferior foragers and unable to compete for the main prey groups in demand. This difference in foraging ability may indeed go part way in explaining why they are not breeding. Further studies identifying the role of jellyfish in Adélie diet, as well as differences between size and species of other prey consumed by adults and chicks, would provide more information on prey preferences during provisioning and self-feeding.

## Data accessibility

Data can be accessed from the Australian Antarctic Data Centre: (http://dx.doi.org/10.4225/15/5514A9C54A8EC).
